# The County Medical Societies of Texas

**Published:** 1887-12

**Authors:** 


### THE COUNTY MEDICAL SOCIETIES OF TEXAS.

It is a source of much gratification to observe the activity of the various county medical associations, and the interest taken by the members in the meetings, especially when we look back two years, and reflect that there was scarcely such an organization in the State. The members frequently ride ten and twenty miles, and they either read a paper, or engage in the discussion of some subject of common interest; and men who had never before attempted to put their observations on paper, are now known as good writers, their papers being, if not polished literary productions, in all cases, are of practical value.

We wish to encourage this, and to see it become more general; and to this end we invite members of the several county societies to send us their productions, for publication, no matter if they be only clinical notes; we will put them in shape. Give the brethren the benefit of your *ideas*, your observations and experience: You can form no idea of the value of such contributions; they are read, and they have their influence. We solicit contributions to every department of the JOURNAL, and especially invite secretaries to send in reports of their meetings. Extra copies of the JOURNAL containing these papers and reports will be sent the contributors, or the secretaries, free of charge, for circulation in their neighborhoods.

